# Managing changes in the environment of human–robot interaction and welfare services

**DOI:** 10.1007/s10799-023-00393-z

**Published:** 2023-03-14

**Authors:** Outi Tuisku, Satu Parjanen, Mirva Hyypiä, Satu Pekkarinen

**Affiliations:** 1 School of Industrial Engineering, Tampere University of Applied Sciences, Tampere, Finland; 2 School of Engineering Science, Lappeenranta-Lahti University of Technology LUT, Lahti, Finland

**Keywords:** Service robot, Change management, Welfare services, Human–computer interaction, Human–robot interaction

## Abstract

The purpose of this study was to investigate decision-makers’ views on changes that robotics will create in welfare services. The purpose was also to discover what the opportunities and challenges are in human–robot interaction during these changes and how to manage these changes. As a research method, an online survey was used. The survey was sent to Finnish decision-makers (N = 184). They were divided into three groups: Techno-positive (n = 66), Techno-neutral (n = 47), and Techno-critical (n = 71). According to the results, more than 80% of the respondents saw that robots can offer support in existing work tasks, and more than 70% saw that the robots can do existing tasks. The most often mentioned challenges were the reduction of interaction and the reduction of human touch. Further, there are various knowledge needs among the respondents. Most of the knowledge needs were not based on the technical use of the robots; rather, they were quite scattered. The results suggest that successful use and implementation of robots in welfare services require a comprehensive plan and change agents. This study suggests that techno-positive people could act as change agents, assisting in implementing the changes. In addition, to manage change in the welfare services it is essential to improve the quality of the information, solve the resistance to change, create organizational awareness, and understanding, and establish a psychological commitment to change the processes.

## Introduction

*Robots are replacing humans*. *Robots are taking over the world*. These are notions that human–robot interaction (HRI) researchers have become used to hearing, ideas that are mentioned quite often in public. What is often forgotten in the discussion is that robots have been used in industry since the 1960s in the assembly lines of General Motors [see, e.g., 1]. The industrial robots have not entirely replaced human work but have changed people’s work tasks [2]. Also, in services, digitalization has had many impacts on how services are organized. Human work is still needed, but new ways of human workers collaborating with technology are sought [e.g.,^3^]. Human and robot capabilities are most productively harnessed by systems where they function collaboratively in ways that complement each other to solve the issues at hand. For example, in the context of intelligent transport systems, HRI may enhance the mobility of human beings and goods, safety increase, traffic congestion reduction, and effective management of incidents [4]. However, the HRI can be complicated and there are many unresolved issues. For example, Etemad-Sajadi et al*.* [5] recognized several ethical concerns that influence the intention to use a service robot including trust and safety, privacy and data protection, and human workers replacement. Also, Mukherjee et al*.* [6] highlight that trust plays an essential role in the approval of robots in the hospitality sector. If the employees are not able to trust the robots, it generates change resistance and creates insecurity among the employees. The core question in the future, particularly in welfare services, concerns the division of labor between humans and robots [See 7]. Here, welfare services mean social and healthcare services, such as primary and specialized health care and elderly care. Well-defined roles between human beings and technology are particularly important in this field, where the tasks have traditionally been based on human touch and interaction but are becoming increasingly affected by digitalization.

The change has not come overnight but has required planning work and changes in leadership practices as well [e.g., 8]. The use of robots poses a challenge to society as a whole. In welfare services, many different types of robots have been utilized or piloted. For example, transportation robots have been used to carry medicine or heavy things [9], socially assistive robots have been utilized to instruct exercises in physiotherapy [e.g., 10], robot companions have been used for therapy purposes [11], and so on. A wide variety of robotic support systems exists, either in a piloting phase or in commercially available forms [12]. Robots bring plenty of opportunities but also potential threats, and there is constant discussion about whether robots are welcomed in care, and if they are, how, for whom, and in what areas [13].

In welfare services, the stakeholder network around robots includes many types of stakeholders who represent the different interests and fields of expertise and who contribute to value creation in the network. For example, the network may include companies that manufacture robots, companies that sell robots, researchers in various fields who develop robots and determine their abilities, the people responsible for the acquisition of robots, care services administrators and managers, and care workers. Legislation also plays a role in relation to robots by controlling the funding for acquisition, determining the responsibilities of service providers who use robots, and regulating health technologies [14]. Thus, in this study, these stakeholders in the network are hereafter called decision-makers.

**Fig. 1:**
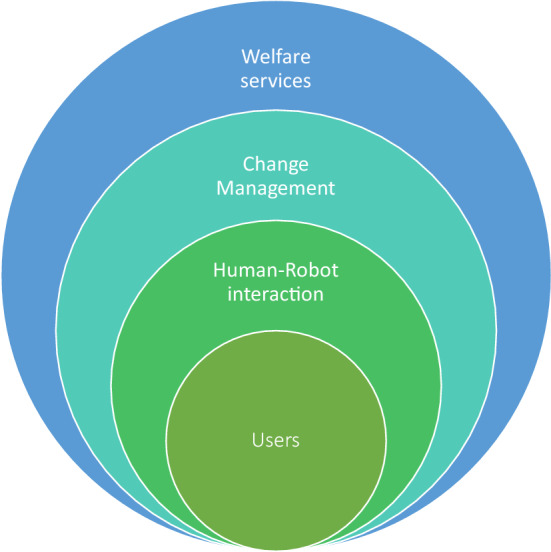
Bridging the key concepts of the theoretical background

The use of robots can be seen as involving three aspects in welfare services. First, welfare services are a unique environment for a robot, due to its nature (see more in the chapter, Human–robot interaction in welfare services). Before a client of welfare services can interact with a robot, many steps need to be taken. Second, those at management levels, who are in charge not only of the acquisition of new technological solutions but also of providing opportunities for care workers to obtain knowledge and competences, face a situation concerning change management (see more in the chapter, Managing changes in human–robot interaction in welfare services). The third aspect is the HRI, how the robot is used, how it can change the services, and so on (see more in the chapter, Human–robot interaction in welfare services). All three aspects are in connected to one other, and none cannot exist without the others, as can be seen also in Fig. 1.

The intertwining aspects become essential when discussing management in welfare services. Related to the view that the users are increasingly the focus and coproducers of services [see e.g., 15], the users play a central role here. This also means that the intertwining changes in all three types of interaction experience effect their roles as well. In this study, both the clients of the services (end users) and the care professionals (who use technology in their work) are considered as “users.”

Purpose of this article is to investigate how to manage change in a turbulent environment such as that in welfare services. To the best of our knowledge, the opinions of these decision-makers on changes to welfare services, what challenges robots bring, and what kind of education is needed, have not thoroughly been studied. However, these people’s opinions are very important in the context of HRI and welfare services because they are the ones who should be part of the introduction of robots to welfare services. With the knowledge gained about the current situation, is it possible to educate decision-makers to understand the processes that the robots will change?

The aim of the study is to be fulfilled by investigating decision-makers’ views on the changes that robotics will create in welfare services. An online survey was conducted that was sent to decision-makers that were envisioned as being part of the welfare services robotics ecosystem see also [16]. The aim is to discover three aspects of the welfare services and HRI: (1) How the decision-makers envision the future of robotics and welfare services, (2) what kind of challenges and opportunities they see, and (3) how they envision the change in welfare services with the robotics. By answering these research questions several theoretical and practical implications are produced. In this study, HRI is connected to change management theories, and it indicates that in complex organizations the methodological framework for change management may not be strictly followed. In addition, this study sheds light on how to implement the change successfully by taking the possible human resistance into account.

This paper is structured as follows. After this introduction, the conceptual foundation is provided including the human–robot interaction in welfare services and the framework of change management. The empirical part of the paper presents the context of the study, the data and its analysis. The next section introduces the results of the quantitative and qualitative analysis. This is followed by discussion about the results, theoretical and practical implications, directions for further research and conclusions.

## Background

### Introduction to care robots

A robot is defined as “a physical object that can move and potentially manipulate the physical world, with at least some degree of autonomy” [17]. Because there are many different types of robots, they can be further divided into two categories: industrial robots and service robots [18]. Service robots are those “that perform useful tasks for humans or equipment excluding industrial automation applications” [17]. There are many different types of service robots, and in fact, Bedaf et al*.* [19] show that 109 service robots have been developed, but only six of these are commercially available. Personal care robots, in turn, are defined as service robots that performs actions contributing directly towards improvement in the quality of life of humans, excluding medical applications [20].

Fosch-Villaronga and Drukarch [21] in their literature review of health care robots, categorized health care robots as (1) surgical robots, (2) (socially and physically) assistive robots and (3) healthcare service robots. Surgical robots are service robots supporting surgeons during surgical procedures. They operate in acquisition and analysis of information, division of surgical trajectories or plan of actions or execution of surgery. Assistive robots are service robots that assist users through physical or social interaction. Socially assistive robots provide direct support to users and include robots that may be used for therapy purposes for instance to people with autism, dementia, or neuro-development disorders, and care robots providing e.g., companionship or basic assistance. Physically assistive robots include e.g. exoskeletons, walking aids, feeding robots, smart wheelchairs, orthoses, and robotic prostheses. Healthcare service robots include, for instance routine task robots, which are autonomous mobile robots that assist medical staff with delivering food and medicine, carrying linens, pushing beds or transferring lab specimens. Healthcare service robots also include telepresence robots for creating as sense of physical presence in the remote place as well as disinfectant robots, the interest towards which has been intensified due to the COVID -19 pandemic [21].

While the definition of a robot suggests that robots should move with some autonomy, in practice, this is not possible in every case, and there is a need for human operator. In this case, the question is not about a robot, but a robotic device [22]. Thus, the interaction between robot and human, HRI, is an essential aspect to consider for the use of service robots to be more widely accepted [23]. This is especially true in welfare services, where different types of service robots differ considerably from each other, based on their appearance and, therefore, on their tasks.

### Human–robot interaction in welfare services

HRI is about the knowledge of how the robot is controlled, how the robot interacts with the client/controller, how the introduction of robots changes the services provided, what the robot looks like, and so on. In addition, it should be taken into consideration that robots do not operate in a vacuum, but they interact with humans in different ways and there are many ethical and legal questions surrounding their design and implementation [24–26]. One ethical question is related to trust. Aroyo et al*.* [27] remind that trust is highly context-dependent, varies among cultures, and requires reflection on others’ trustworthiness, appraising whether there is enough evidence to conclude that these agents deserve to be trusted. In addition, more research is needed on what happens when too much trust is placed in robots and autonomous systems. The introduction to robots should take these factors into account [28] as well as, for example, the funding issues. However, these issues are not entirely straightforward, due to differences between robots. Because of the differences, while one might master the use of one robot, that might not be the case with another robot. Thus, the HRI should be designed to be as natural as possible, especially in such human-centric field as welfare services.

Here, welfare services mean social and healthcare services, which, in Finland, are of the public sector’s responsibility. To be more specific, the responsibility for organizing such services lies with local government, the municipalities. The Finnish healthcare system provides comprehensive health care for all citizens. The funding for public healthcare services is mainly handled via the Finnish tax system, and charges to service users are low [16, 29]. In welfare services, cost is an important criterion, and in a discussion about robotics, one of the first arguments against it relates to funding or other monetary issues [e.g., 30]. Yet it could be that robotics or other digital solutions might not even add additional costs or tie up additional recourses. The consideration of *opportunity costs,* which follow from choosing a certain alternative instead of another, is central when examining social and health care services [31].

Digitalization plays a major role in renewing welfare services, particularly in meeting the anticipated sustainability gap in elder care services [16, 32–34]. However, balancing economics on the one hand and social and human sustainability on the other is not an easy task in the present economic situation, and various confrontations may appear while balancing human orientation with increasing efficiency [35]. High hopes have been placed on technological innovations such as e-health, various types of monitoring, home automation, robotics, and simpler applications [36], and the Finnish government has adopted strategies to enhance the digitalization of public services [37]. Services can be produced in a new manner, and service processes can be optimized with the help of digital tools, with the aim of supporting both users and professionals. Thus, digitalization means both hardware (different kinds of technological devices) and software (information systems) as well as combinations of the two, in addition to human factors and practices.

However, one must be careful with “techno-solutionistism,” where, for instance, technology is widely perceived to provide the means of solving the “grand challenge of demographic ageing” [see 34]. Neven [38] and Peine et al*.* [34] note that gerontechnological innovations, e.g., service robots, are often embedded in a “triple-win narrative,” in which policy-makers, technology developers, and older citizens are said to benefit equally from these innovations. However, if the various human aspects and user contexts are not involved, e.g., if older technology users are given only a stereotypical identity as passive recipients and are not viewed as active agents, it may lead to a triple loss instead of a triple gain [34]. The influence of digital technologies, including service robots, on clients and care-service personnel holds implications for integrating technological innovations into care [10, 39, 40]. Therefore, the management’s role is particularly important in this integration process.

Bringing robotics to fields that are closer to humans has naturally raised some doubts. This is especially true when introducing robots into welfare services, where their use is not without problems. They bring up resistance on many different levels. For example, public opinion is often negative towards the use of robots in welfare services [30], even though the public has little or no experience using robots. People who use (or should use) robots in welfare services also have negative attitudes towards them [41]. These people worry, for example, that robots will replace the human workforce in care [42]. Yet although surgery robots are widely used, they have not replaced surgeons but serve as their tools [43]. The case is usually that when care workers have experienced use of a robot, their attitude becomes more open to the possibilities of such usage [e.g., 10, 44]. Of course, integrating or trying to integrate new technological devices/services in welfare services is often not problem-free. It is often the task of a care manager or a decision-maker at another level to introduce the technology and have an interest in acquiring new technological solutions.

Attempts to introduce robots into welfare services have not been very successful so far [45]. The general attitude towards care robots has tended to be more negative compared to other uses of robots [e.g., 46, 47]. For example, Turja et al*.* [48] show that general views on robots are more positive among the Finnish population compared with health care professionals, and practical nurses stand out as having the most reserved attitudes towards robots. However, positive attitudes towards robots seem to correlate with the amount of experience with robotic devices [e.g., 12, 48, 49].

Ideally, the acquisition and utilization of robots in welfare services should follow Rogers’ [50] diffusion of innovation model: First, robots are adopted by a relatively small group of “innovators” and “early adopters,” in this case, the decision-makers. The increased knowledge about the possibilities of robots should then attract more users, who form the “early majority,” in this case, the users. Once there are plenty of users, robots will be seen as a natural part of welfare services, which will lead to the “late majority” accepting the robots into regular use. Finally, the small group of “laggards” also accept the robots. Thus, the acquisition might depend on the manager’s skills and expertise in using the new solution but also on his/her interest in leading the change and acting as a role model by utilizing the robot in his/her work.

While Rogers’ [50] categorization of users tackled about actual adoption of innovations, there have also been studies which tackle also general attitudes towards technology and readiness to use them, which is Hanesova et al*.* [51] talk about techno-positive and techno-negative (techno-sceptic) attitudes. A more refine categorization has been made by e.g., Tomchyk et al*.* [52], who studied the opinions expressed by Polish and Czech students, have distinguished four categories in this sense: techno-optimist, techno-realist, techno-pessimist and techno-ignorant. Techno-optimists have an enthusiastic attitude towards technology and are not afraid of unfamiliar technological solutions. Techno-realists are characterized by a certain distance towards new technologies, which does not mean a reluctance to modify their own style of working according to technological progress, but a careful, conscious openness to new possibilities which they carry. They are not interested in technological inventions and are neutral towards changes in that area. Techno-pessimists are characterized by a negative attitude towards new technologies and a belief that they are useless (in the moderate option) or unfavorable for human development and functioning. Techno-ignorant are, characterized by a lack of involvement in learning about new media. Such an attitude will be expressed through isolation from new information and communication technologies, the avoidance of learning about them and expressing opinions about them [52].

### Managing changes in human–robot interaction in welfare services

The changes in work life are permanent, and therefore, there is a growing demand for new tools, new knowledge of and approaches to management, a new steering of operations, and a new decision-making culture [53–56]. However, there is lack of published literature concerning frameworks that are particularly successful for change in the health care sector [57, 58]. Despite the era of digitalization, leadership and management processes are still led by people, especially in the health care sector, and new technologies and information require interpretation and continuous dialogue [53, 54]. According to Niiranen et al.[56], there are very different and somewhat conflicting expectations of managers and management work at different levels in the social and health care sector*.* The cultural challenges are enormous, and therefore, privacy concerns will only become more significant. But the underlying trends in any field, such as technology, health care, and business payoff, are unmistakable [45, 53].

Change management has been an active research area for several years; take, for example, Kurt Lewin’s unfreeze–change–refreeze steps [59], Kotter’s 8-step change model [60], and Schein’s “Lewinian” model of change/learning [61]. In this study, the perspectives of the decision-makers in change management in various industries as well as Varkey and Antonio’s [58] methodical framework are presented. The aim is to promote change initiatives with respect to HRI in welfare services. Varkey and Antonio’s [58] rather practical approach was chosen for this study because of its focus on the people in the health care organizations; the social nature of their study also supports previous research on robotics in Finnish welfare services [62].

Change management is an action or process undertaken to smoothly transition an individual or group from the current state to a future desired state of being. Thus, successful change management is divided into the following key steps in Varkey and Antonio’s [58] framework: (1) assessing readiness for change, (2) establishing a sense of urgency, (3) assembling the steering team, (4) developing an implementation plan, (5) executing a pilot, (6) disseminating change, and (7) anchoring the change within the organization.

Pekkarinen et al*.*’s study [62] also exposed three main factors that hinder the introduction of robots in welfare services: the care culture, resistance to change, and a fear of robots. In other words, the hindering factors related to robot use in welfare services are largely attitudinal and mostly related to mindset-issues and path-dependent operational cultures rather than to for instance technological limitations [62].

Previous literature suggests using change agents in organizational changes [e.g., 63, 64]. A change agent needs to successfully construct rationales to justify the desired organizational change to convince other employees that they should not resist it [65]. There are several reasons why change is resisted. Oreg [66] found that both personality and context were significantly associated with employees’ attitudes towards a large-scale organizational change. Other reasons for resistance include a lack of motivation, uncertainties, an increased workload, and concerns about competence [67].

## Research methodology

### Context of the study

In Finland, one of the duties of the public sector is to take care of the health and well-being of the population. This is done in part by arranging for welfare services, meaning here social and health services, the responsibility for which falls to local government, that is, the municipalities. Municipal social welfare and health care services, implemented with government support, form the basis of the social welfare and health care system. Health services are divided into primary health care and specialized medical care. Primary health care refers to the municipally arranged services, which include monitoring the health of the population; promoting well-being and health; and prevention, diagnosis, and treatment of diseases, in particular public health diseases. Primary health care services are provided at municipal health centers. Specialized medical care refers to secondary and tertiary health care, provided by experts in medical (or dental) specialties. To a large extent, specialized medical care is performed in hospitals, but it is also offered as consultations with primary health care [29].

Private companies also provide services alongside the public sector. Finland has a wide range of social welfare and health care organizations, providing services both free of charge and for a fee. The purpose of the policy directed by the government is to ensure that the social and health care client fees are kept to a reasonable level and that they are not an obstacle to using the related services. Social and health services are generally either free of charge or are provided for a client fee, which, depending on the service, is either fixed or depends on the client’s income and family relations [29].

### Survey

An online survey for different stakeholders in Finland was conducted in the spring of 2017. The survey was sent to a wide variety of decision-makers in the welfare services robotics ecosystem [62] with a judgement sampling technique, i.e. the respondents were selected from the identified relevant decision-makers in the field of care robotics, which included members of parliament, ministries, enterprises in the field of robotics, associations, research institutes, and municipalities and hospital districts. Our selection was based on the notion of welfare services as a sociotechnical system [14, 35 based on 68] consisting of the industry, infrastructure, and service structures producing health-care products and services, the products and services themselves, as well as the related policies and markets. On the basis of these elements of the sociotechnical system, we identified the stakeholders to include in the study.

For the member of parliament, the survey was sent to their personal parliament email address. For the enterprises and associations, the survey was sent to the general email address, that was found from the webpages of the company. For the research institutes, the survey was sent to such researchers that work within the field of robotics in Finland. For the municipalities and hospital districts, the survey was sent to registry offices with a letter that asked that the survey invitation be forwarded to related people, such as the director of elder care, the director of social services or similar persons as well as to the members of municipal councils. The total number of people contacted was approximately 1000, and the survey was open for three and a half weeks in February–March 2017. Accordingly, the stakeholders of the study were identified to be the actors of this socio-technical system: namely stakeholders from the industry, services and policy-making.

### Content of the data

The survey consisted of the following elements: background information, general questions about robotics, robotics issues in welfare services, and questions related to care robots. In total, the survey consisted of over 50 questions. The questions were asked in Finnish, the respondents’ native language, but were translated into English for this article. The survey questions were designed by the authors, based on previous research [12, 30, 32, 33, 46, 69, 70]. The study followed the ethical principles outlined in the Declaration of Helsinki of 1975, as revised in 2000 and 2008. In this paper, 9 questions were analyzed, which were divided into three themes based on the topic of the questions in the survey. The three themes were: (1) Future of robotics and welfare services, (2) Challenges and opportunities in the use of robots, and (3) Change in welfare services via robots.

The theme “Future of robotics and welfare services” theme was investigated with five different claims:

- Robotics will reduce the workload of the welfare services staff.
- Robotics will improve the quality of welfare services experienced by the client.
- Robotics will degrade the quality of welfare services.
- Robotics will replace people’s work in producing welfare services.
- Robotics will not play a significant role in welfare services.

These claims are based on literature: on the one hand on the expectations that robotics and other technologies will help to tackle the challenges of ageing societies and facilitate the care burden [32, 33], while on the other hand on the fears that robots will replace people and human touch in care and thus degrade the quality of care [69, 70]. The formulation of the claims is also influenced by Eurobarometer survey [46] claims, but these are not use as such. These claims were answered using a 5-point scale that varied from 1 (i.e., totally agree) to 5 (i.e., totally disagree). The 3 response represented the neutral opinion. The claims were shown to the respondent one by one.

The theme “Challenges and opportunities in the use of robots” theme was investigated with two different multiple-choice questions:

- What challenges do you think are related to the use of robots in welfare services?
- What opportunities do you think the use of robotics brings to the workplace?

The respondents were shown a list of choices, and they were allowed to mark down as many choices as they wished.

The theme “Change in welfare services via robots” theme was investigated with two different open questions:

- How do you evaluate that the welfare services will change, from the client’s perspective, with the use of robots?
- What kind of new skills and training do you think are needed when the use of robots increases?

In these questions, the respondents were able to write as long an answer as they wished.

### Respondents

Altogether, 184 people responded to all the questions discussed in this article. The respondents were divided into three groups: techno-positive (35.8% of the respondents), techno-neutral (25.6%) and techno-critical (38.6%) based on their response to the claim, “Robotics will resolve the problems related to the sufficiency of welfare services.” These categories were formulated based on earlier research, for instance by Hanesova et al*.* [51] and Tomczyk et al*.* [52]. The “Techno-positive group” consisted of respondents who totally agreed or agreed with the claim (n = 66); the “Techno-neutral group” consisted of respondents who responded neutrally (n = 47); and the “Techno-critical group” consisted of respondents who disagreed or totally disagreed with the claim (n = 71). The demographics of the respondents are presented in Table 1.

**Table 1:** Demographic data of the respondents

		Techno-positive	Techno-neutral	Techno-critical
Gender	*Female*	44	38	54
	*Male*	22	9	17
Age division	*Under 25*	0	0	0
	*26–35*	3	3	5
	*36–45*	10	7	11
	*46–55*	17	17	25
	*56–65*	26	18	23
	*Over 65*	10	3	7
Educational background	*Comprehensive school*	2	0	2
	*Vocational school*	3	2	2
	*Secondary school graduate*	1	2	0
	*Bachelor’s degree*	14	7	12
	*Master’s degree*	36	19	44
	*Postgraduate degree (PhD or equivalent)*	8	15	8
	*Other*	2	2	3
Work in societal sector	*Municipal*	35	23	41
	*Private*	4	5	4
	*Third*	16	11	14
	*State*	5	6	6
	*Other*	6	3	5
Knowledge on robotics	*Excellent*	4	0	1
	*Good*	18	6	12
	*Mediocre*	16	20	19
	*Satisfactory*	14	10	16
	*Weak*	14	11	23

### Data analysis

The numerical data was analyzed with one-way analysis of variance (ANOVA), with the response group as the dependent factor, for each of the questions separately. Bonferroni corrected pairwise analysis was used for post-hoc pairwise comparisons. Cronbach’s Alpha was used for reliability analysis. The data was analyzed using IBM SPSS Statistic software, version 26.

For the multiple-choice questions, where participants were able to mark down as many responses as they wished, each of the options was treated as a single variable in the analysis. The questions were coded so that if a participant selected an option, the response was marked as 1, and if the participant did not select an option, it was marked as 0.

The qualitative data was analyzed by means of quantitative and qualitative content analysis [71]. The data was first read through many times to comprehend the content. Then the comments with similar content were grouped together, and the category was given a descriptive name. The number of comments for each category was calculated to show how the responses were divided. These categories were again checked with the comments and the entire dataset for relevance and for ensuring the reliability of the data. In addition, the present study utilized the triangulation of data (quantitative and qualitative data) and investigators in order to understand a complex phenomenon and to increase the quality of the study.

## Results

The results are divided into three themes introduced in the method section: the “Future of robotics and welfare services,” “Challenges and opportunities in the use of robots,” and “Change in welfare services via robots.” The logic to arrange the result section is to move from more general theme to more detailed theme to give the overall image for the reader.

### Perceptions of the theme: future of robotics and welfare services

The respondents were asked to evaluate the status of welfare services with five claims about the quality of the welfare services when they include the use of robots.

**Fig. 2:**
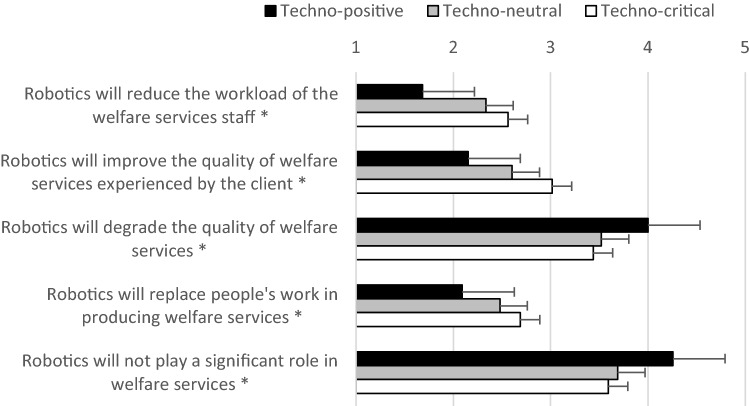
Mean values for each claim (1 = totally agree, 3 = neutral, 5 = totally disagree). The claim “Robotics will resolve the problems related to the sufficiency of welfare services” was used for grouping the respondents, and thus, the responses are evenly divided for that claim. The statistically significant claims are marked with *

Figure 2 shows the mean values (+ standard error) for each claim (Cronbach’s Alpha 0.51).

The one-way ANOVA showed a statistically significant main effect of the group for all six claims; Robotics will reduce the workload of the welfare services staff F(2,184) = 23.87, *p* < 0.001, Robotics will improve the quality of welfare services experienced by the client F(2,183) = 16.24, *p* < 0.001, Robotics will degrade the quality of welfare services F(2,184) = 7.17, *p* < 0.001, and Robotics will not play a significant role in welfare services F(2,184) = 11.87, *p* < 0.001. These results indicated that respondents in each of the groups clearly had different ideas of what kind of role robotics would have in welfare services in the future. The Techno-positive group considered robots to be a possibility in future welfare services, while the other two groups were somewhat more resistant towards robots. The post-hoc pairwise comparisons are shown in Table 2.

**Table 2:** Post-hoc pairwise comparisons for the five claims

Claim	Groups	Mean difference	*p*-value
Robotics will reduce the workload of the welfare services staff	Techno-positive < Techno-neutral	0.65	*p* < 0.001
	Techno-positive < Techno-critical	0.88	*p* < 0.001
	Techno-neutral = Techno-critical	–	ns
Robotics will improve the quality of welfare services experienced by the client	Techno-positive < Techno-neutral	0.45	*p* < 0.05
	Techno-positive < Techno-critical	0.86	*p* < 0.001
	Techno-neutral < Techno-critical	0.41	*p* < 0.05
Robotics will degrade the quality of welfare services	Techno-positive > Techno-neutral	0.48	*p* < 0.05
	Techno-positive > Techno-critical	0.56	*p* < 0.01
	Techno-neutral = Techno-critical	–	ns
Robotics will replace people’s work in producing welfare services	Techno-positive = Techno-neutral	–	ns
	Techno-positive < Techno-critical	0.59	*p* < 0.01
	Techno-neutral = Techno-critical	–	ns
Robotics will not play a significant role in welfare services	Techno-positive > Techno-neutral	0.57	*p* < 0.01
	Techno-positive > Techno-critical	0.67	*p* < 0.001
	Techno-neutral = Techno-critical	–	ns

From the pairwise comparisons (in Table 2), it is evident that the Techno-neutral and Techno-critical groups did not differ from each other for most of the claims. The only statistically significant difference between these groups was in the claim, “Robotics will improve the quality of welfare services experienced by the client,” where the Techno-neutral group agreed more with the claim.

### Perceptions of the theme: challenges and opportunities in the use of robots

**Fig. 3:**
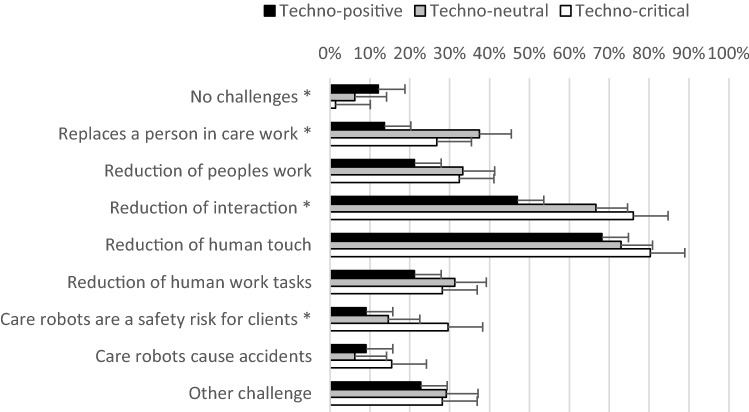
Mean values of responses to the question, “What kind of challenges, in your opinion, are related to the use of robots in welfare services?” The statistically significant factors are marked with *

The challenges related to the use of robots in welfare services were investigated with one multiple-choice question: What kind of challenges, in your opinion, are related to the use of robots in welfare services? In this question, one participant was allowed to mark as many choices as they wished. On average, one respondent gave 2.79 answers (Cronbach’s Alpha 0.52). Figure 3 shows the mean values (+ standard error) for each of the categories.

One-way ANOVA showed a statistically significant main effect of the group for four of the factors: No challenges F(2,184) = 3.31, *p* < 0.05, Replaces a person in care work F(2,184) = 4.48, *p* < 0.05, Reduction of interaction, F(2,184) = 6.75, *p* < 0.01, and Care robots are a safety risk for clients F(2,184) = 5.31, *p* < 0.01.Table 3 shows the post-hoc pairwise comparisons for the statistically significant factors.

**Table 3:** Post-hoc pairwise comparisons for statistically significant factors

Question	Groups	Mean difference	*p*-value
No challenges	Techno-positive = Techno-neutral	–	ns
	Techno-positive > Techno-critical	0.11	*p* < 0.05
	Techno-neutral = Techno-critical	–	ns
Replaces a person in care work	Techno-positive < Techno-neutral	0.24	*p* < 0.05
	Techno-positive = Techno-critical	–	ns
	Techno-neutral = Techno-critical	–	ns
Reduction of interaction	Techno-positive = Techno-neutral	–	ns
	Techno-positive < Techno-critical	0.29	*p* < 0.01
	Techno-neutral = Techno-critical	–	ns
Care robots are a safety risk for clients	Techno-positive = Techno-neutral	–	ns
	Techno-positive < Techno-critical	0.21	*p* < 0.01
	Techno-neutral = Techno-critical	-	ns

In the comment field for the response “Other challenges,” security issues were particularly highlighted related to information security and technical problems that might cause unwanted consequences, for example a client not getting the care they need or being given the wrong dose of a drug. This highlights the importance of good planning and design. One respondent (Techno-positive) also stated that inexperienced users in particular might experience fear, confusion, and anxiety when using robots.

**Fig. 4:**
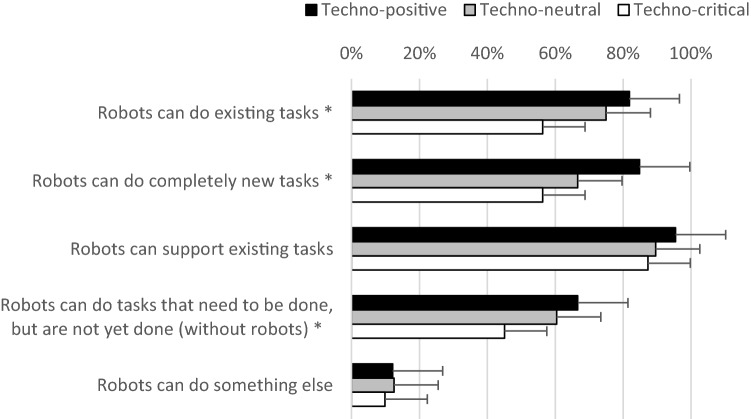
Division of responses for the question: What opportunities do you think the use of robotics brings to the workplace? The statistically significant factors are marked with *

The opportunities were investigated with the following question: What opportunities do you think the use of robotics brings to the workplace? In this question, one respondent was allowed to mark as many choices as they wished. On average, one respondent gave 2.98 answers (Cronbach’s Alpha 0.65). Figure 4 shows the mean values (+ standard error) for each category.

One-way ANOVA showed a statistically significant main effect of the group for the three factors; Robots can do existing tasks F(2,184) = 5.94, *p* < 0.05, Robots can do completely new tasks F(2,184) = 7.01, *p* < 0.01, and Robots can do tasks that need to be done but are not yet done (without robots), F(2,184) = 3.51, *p* < 0.05. The post-hoc pairwise comparisons are shown in Table 4.

**Table 4:** Post-hoc pairwise comparisons for statistically significant factors

Question	Groups	Mean difference	p-value
Robots can do existing tasks	Techno-positive > Techno-neutral	1.14	*p* < 0.001
	Techno-positive > Techno-critical	2.36	*p* < 0.001
	Techno-neutral > Techno-critical	1.23	*p* < 0.001
Robots can do completely new tasks	Techno-positive = Techno-neutral	–	ns
	Techno-positive > Techno-critical	0.28	*p* < 0.001
	Techno-neutral = Techno-critical	–	ns
Robots can do tasks that need to be done, but are not yet done (without robots)	Techno-positive = Techno-neutral	–	ns
	Techno-positive > Techno-critical	0.22	*p* < 0.05
	Techno-neutral = Techno-critical	–	ns

In the comment field, “Robots can do something else,” some respondents mentioned that robots are able to do tasks that are unpleasant or dangerous for people. The respondents also considered that robots are able to assist in complex decision-making, facilitating a disabled person living at home by observing the environment, filling out forms, or searching information in the internet as well as monitoring security. One respondent (Techno-neutral) was worried that robots could do illegal things such as crimes and sharing disinformation, and according to one respondent (Techno-critical), the robots would take all humans’ jobs and were totally useless devices.

### *Perceptions of the theme: change in welfare services *via* robots*

This topic was investigated with two open questions. First, it was asked how respondents evaluated how welfare services would change from the viewpoint of the client when robots were introduced to welfare services. Table 5 shows the responses within the groups.

**Table 5:** Grouping of responses for open question on how welfare services will change when robots are introduced

	Comments in open answers	Amount		
#		Techno-positive	Techno-neutral	Techno-critical
1	Improving the quality and effectiveness of services	14	2	3
2	Improved access and accessibility to services	23	5	17
3	Individualization of services and improved privacy	2	1	1
4	Economy (cost-effectiveness, service price calculation)	4	2	-
5	Security (accuracy, timeliness, reliability, accuracy)	11	3	3
6	Changing the work of nurses (shifting routine tasks to robots, enabling encounters with clients)	12	5	11
7	Strengthening the clients’ independence, which, among other things, enables independent living at home	12	4	12
8	Improved contacts and communication (between client and caregiver, contacts with experts, relatives, friends)	5	7	1
9	Improving service efficiency and speed	4	5	5
10	Increasing clients’ well-being, which has effects, for example, on mental health	5	2	4
11	Information technology/artificial intelligence contributes to processes (diagnostics, decision-making, foresight)	2	3	7
12	Decreased human contact and humanity, loneliness, insecurity (life narrows, impoverishes, facelessness)	9	8	13

In the Techno-positive group, the responses mainly referred to improving the quality and effectiveness of services, improving their availability and accessibility, increasing security, and strengthening customer engagement. According to the respondents, there will be “more better-quality services despite reduced resources, because nurses will have enough time to do more than just that is absolutely needed” or “the security would probably increase, the availability of some services would be better, well-being will be better”. The answers were more positive than in the other groups, although many comments raised concerns about the reduction in human contact. Only one negative answer was given in this group.

In the Techno-neutral group, the entries were more scattered, but most frequently mentioned was the improvement of contacts and communication. The answers clearly emphasized that if things went well, good things could happen but that it was also possible that things would be “pushed,” and the situation might not improve or might even get worse. One example of this kind of answer is the following: “It [the use of robots] could make life easier and release the family members’ and nurses’ time more to human interaction. There is a risk that clients, family members and nurses will waste their time on battling with unfunctional and unfinished robots and that because of savings and efficiency demands human presence and contact are being replaced by robots. That's when loneliness, your sense of insecurity and needlessness may increase.” There was the reminder that robots did not replace the caregiver and that robotics were not suitable for all services as one respondent highlighted: “…some services will be automatized because of the robots, but robots are not suitable for every service, there is a need for people and interaction”. They also mentioned that the ability of the client to accept the change was essential and that functional systems were needed. The issue was viewed more broadly, both positively and negatively, and it was demonstrated that there is no single answer to this question.

The Techno-critical group mentioned improvements in accessibility like “Robots will create possibilities for welfare services, for example, giving services at home” or “hopefully the accessibility of services will be easier and faster”. This group also mentioned changes in nurses’ work like “Routine tasks are transferred, where applicable, to robots.” or “Humanity disappears from nursing”. In addition, respondents expressed concern about reducing the workforce like in following answer: “I am afraid, that in the worst case, the robots will make it possible that there will be less staff and the quality of the nursing will decrease”. This group also clearly emphasized that it was possible to collect information that could then be utilized in different processes. In the other groups, it was primarily mentioned that the robotics could be used to monitor a patient and, thus, allow one to know when, for example, a doctor should be called. In this group, more attention was paid to what could be done with this information (service planning, information management, anticipation of service needs). This group also mentioned more individual negative effects, such as the risk of the robot replacing humans, the suffering of quality, equipment and IT skills, electricity and energy consumption, and the fear that robots were just a way to save costs.

Next, the perspectives of the future were also investigated, particularly what kind of new education and training would be needed when the use of robots became more common. Table 6 shows the responses according to the different types of education needed. The responses in the different groups did not differ from each other, and thus, the responses are grouped by the topic and are combined to reflect the practical steps of change management [58].

**Table 6:** Grouping of the responses

Education/training	Contents	Change management steps
Acquisition	Purchase and disposal	Assessing readiness for change
Use	Operational logic, utilization targets, implementation, maintenance, error situations, security, customer safety, risks, user orientation, user-friendliness, ability to stand in the position of a customer	Establishing a sense of urgency
Leadership	Developing business processes, guiding free work input to essential work tasks, identifying applications, acquiring robotics	Assembling the steering team
Renewal of work tasks and new tasks	New professions, new ways of doing things, impact, cross-sector collaboration, change in attitude, learning and adaptation coaching, responsibility issues, collaborative work, different experts work on the same issue, appreciation of another’s expertise, reviewing one’s own work as part of change, interdisciplinary competence, work planning and organization, logical thinking, resilience to change, combining technology and well-being, daring to experiment, abstract thinking, creative thinking, courage, interpersonal skills	Developing an implementation plan
Ethics	Strengthening ethical thinking, understanding human ethical choices in relation to robots so that human behavior in relation to robots does not lead to inhuman action, ensuring humanity, clarifying the roles of a human and a robot	Establishing a sense of urgency, Developing an implementation plan
Related to social and health care services	Service control, nursing, diagnostics, device targeting, effectiveness, care technology training	Executing a pilot
Data processing and analysis training	Practical use of information, data integration and transfer between devices and information systems/data banks, correct interpretation of data	Executing a pilot
Computer skills	Software design, design and implementation of robotized entities, coding, understanding of criteria, artificial intelligence	Disseminating change
Training in specific technologies	Positioning technology, artificial intelligence, knowledge and advanced security, virtual modeling, networking skills, sensor technology, monorobot environments, open development environments	Disseminating change
Evaluation of effectiveness	Identifying real effects and benefits, research expertise	Disseminating change
Development of robotics, maintenance and service	User-oriented, user experience, ease of use, popularization, service design	Anchoring the change within the organization
Multidisciplinary education	Education combining technology, psychology and design, education combining technology, aging research, and everyday life	Anchoring the change within the organization

There were no major differences among the different respondent groups; the responses were found to be very similar. The respondents answered, for example, “Acquisition, implementation, maintenance, programming, data networks, security, ethics”, “Safety and ethics of robotic technology, user experience, guaranteeing humanity in the work, finding out the roles of humans and robots”, “technology skills” or “How to use technology as part of your own work, the ability to use robotics as assistive tool”. It was surprising that education of people’s attitudes was emphasized as strongly as the features associated with innovation capacity, such as creative thinking, courage, daring, and resilience to change. In order to support change in decision-making, clear, practical steps are required. For example, the aim of the step of assessing readiness for change is to attract and motivate participants towards the change, and often, the worrying financial investments are identified right at the beginning of the process. On the other hand, after the pilots and dissemination, the change must be anchored to the practices, for example whether new employees or skills are needed, the training of the current staff, and modifications to or disabling of existing technologies to support development work in the welfare services.

## Discussion

### Summary of the key findings

Worldwide, welfare services and the ways people engage with their health and well-being are going through a period of rapid change due to various digital technologies [e.g., 72]. The purpose of this study was to investigate decision-makers’ views on the changes that robotics will create in welfare services. Responses related to the theme of the “Future of robotics and welfare services” quite clearly showed how respondents envisioned the changes in welfare services. The Techno-positive group was the most positive of the groups, which was quite natural, based on the claim that the groups were divided. But it is interesting that people from all different age groups, genders, and educational backgrounds were quite evenly divided in these groups, indicating that age, gender, and educational background do not explain people’s attitudes to robotics. This finding is partly in line with Turja et al*.*’s [48] study about care workers’ readiness for robotization. That study found that readiness was less determined by age, gender, or profession among respondents with firsthand robot experience, while among care workers with no experience with robots, older age predicted a readiness for robotization.

The findings of this study also show that people adopt innovations in different time phases. According to the study of Taylor and Todd [73], the successful implementation of information technology in the organization should be supported by communication, user participation and facilitating conditions. The people with a more positive attitude (Techno-positive) could perhaps be the ones who will tackle the introduction to robots in their organizations and who act as “early adopters” [50]. They may also possibly act as change agents in their organizations, assisting in implementing the change. According to Turja et al*.* [48], potential change agents have a high interest in technology, high robot-use self-efficacy, the perception that coworkers approve of robots, and an optimism that robots will not take peoples’ jobs. Based on the findings of this study, one of the essential tasks of change agents is communication or, as Rogers [50] defines it, sharing information to create mutual understanding about the possibilities and challenges of robotization.

The use of robotics in welfare services includes challenges (the “Challenges and opportunities in the use of robots” theme). The use of robotics in health and elder care has generated much discussion from ethical [69, 74] and employment [75] perspectives, as was also the case in this study. The most often mentioned challenges by far were the reduction of interaction and the reduction of human touch. Similar challenges have also been reported elsewhere [e.g., 10, 69, 70, 74]. In this study, also the information security and possible technical problems were mentioned as challenges that could be solved by good planning and user-driven design. In prior research, it is recommended that the design processes of the robot in a health care context should be co-creative and participatory by nature, involving end-users, nurses and doctors [7, 10, 74, 76, 77]. Actually, Kiesler and Goodrich [78] remind that more user studies with ethnographic research methods will be needed in the field of HRI.

The respondents also considered that robots might reduce human work. Some pointed that robot could reduce the nurses’ workload, meaning that they may have more time to for other activities, but others pointed that robot are going to replace human work gradually. On the other hand, respondents also had concerns that reducing workers had more to do with savings than robots. Fosch-Villaronga et al*.* [74] remind that there are little empirical research how new technologies like robots and artificial intelligence affect the labor market and it is possible that robots have both positive (e.g., increases in productivity) and negative effect (e.g., displacement of workers) on employment. The findings support the fact that balancing human orientation and increasing efficiency is not an easy task in the present economic situation [35]. Interestingly, however, other suggested challenges were not often selected, and overall, those answers were given by less than 40% of the respondents, which is a promising finding. However, in addition to challenges, robots also pose opportunities. The respondents selected many of the listed possibilities, and in fact, more than 80% saw that robots can offer support in existing work tasks, and more than 70% saw that the robots can do existing tasks. Again, the Techno-positive group was the most opportunistic group, answering significantly more often about the possibilities of robots.

The “Change in welfare services” theme was investigated with a different approach than were the “Future of robotics and welfare services” theme or the “Challenges and opportunities in the use of robots.” The questions were open questions where the respondents were able to write down their opinions. For the question about how welfare services will change when the robots come, altogether, 12 topics were found. These topics were related to such things as improving the quality and effectiveness of services. The Techno-positive group was more positive about the change compared to the other response groups. For example, the Techno-critical group brought up individual negative effects, such as the risk of the robot replacing humans, the suffering of quality, equipment and IT skills, electricity and energy consumption, and the fear that robots are just a way to save costs. Thus, according to the respondents, robotization may have both significant negative and positive effects on work in welfare services. This is in line with Smids et al*.* [79] regarding the impact of increasing robotization of the workplace on meaningful work. These respondents conclude that robotization can be either a threat to or an opportunity for meaningful work, and these threats and opportunities should be considered when integrating robots into workplaces.

For the training needs, different options arose. A somewhat surprising result was that most of the knowledge needs were not based on the technical use of the robots; rather, the knowledge needs were quite scattered. For example, the acquisition of robots was seen as important, which entailed many questions, such as where and how to acquire a robot, what kind of robot to acquire, and so on. The acquisition of robots is multifaceted question including client and worker perspectives. For example, from the client perspective the face shape of the robot might have impact on people’s trustworthiness perceptions of the social robot [80] and from the worker perspective the importance of orientation and especially its social aspect are essential [7, 28].

### Theoretical implications

Human–robot interaction is a challenging research field at the intersection of psychology, cognitive science, social sciences, artificial intelligence, computer science, robotics, engineering, and human–computer interaction [81]. Despite the scientific multifaceted perspective, in practice people interact with robots and different technologies almost unnoticed every day in their home and work environments. This study increases the understanding how the challenges and opportunities brought by robotics can be discovered and utilized in welfare services by connecting HRI and change management theories. Robots have not taken people’s jobs so far, in fact, it has turned out quite differently, but robots have an impact on modifying daily tasks [2]. Digitalization and ever-increasing data are growing demand for future working life skills, including health professionals in welfare services. With technology and industry specializing in more identified and human-oriented solutions, the management of changes should evolve as well. Leadership and management theories often support change and guidance at the management level but in this study rather practical approach is applied focusing on social nature of welfare services and people in the health care organizations [e.g., 54, 58, 82, 83]. Yet, health care organizations are more complex than current management theories suggest, and the methodical framework for change management may not be strictly followed in all processes; some of the key steps [58] may be left out, while others may be revisited in a cyclical fashion. The specifics of HRI in welfare services, for example, possible prejudices, technological problems and cyber security and their impacts on work in health care organizations should better acknowledged with management in change processes and theories. Consequently, this study contributes to the scientific discussion concerning frameworks that are particularly successful for change in the health care sector [57, 58]. This study therefore links various perspectives with respect to continual changes, opportunities, management, human–robot interaction, and welfare services, suggesting that to understand the requirements for the transition to the digital era, diversity in scientific paradigms must also be challenged.

### Practical implications

Human resistance to change is a factor that organizations should not underestimate when trying to implement a change. Health care organizations often recognize the existence of resistance in the organization, but they are also often unaware of the reasons behind the resistance, and hence, they cannot efficiently decrease it or prevent it from developing. However, for a change implementation to be successful, human resistance requires the attention of organizations regardless of the type or size of change they are going through.

Communication is very important during a change process [16, 58, 73]. Health care organizations generally acknowledge this, but communication tools and channels are not always efficient in meeting all relevant people or sharing information. Welfare organizations should also realize the importance of repeating information and messages. Feedback and listening skills are also highly valuable, allowing organizations to create a functioning environment of HRI internally as well as externally.

It is also worthwhile to identify aspects of welfare services in HRI that will not change during the transition state and to communicate these to all organization members. For example, these include concerns related to a reduction of human interaction and human touch in general. With respect to job loss, MacCrory et al*.* [84] has stated that AI, machine learning, and so on will never completely replace human interaction or teamwork skills. Digitalization creates new job descriptions and job roles, regardless of sector. The different roles, for example those of early adopters and change agents, are needed not only for the interpretation of data in technology industries but also for the development and deployment of robotics in welfare services.

While seeking to manage change successfully in the welfare services, it is important to improve the quality of information logically and incrementally. Improvements can be achieved related to key decisions by solving resistance to change, creating organizational awareness, and understanding, and establishing a psychological commitment to change the processes [e.g., 48]. Politicians are not leaders but followers; the opinion of the people and the citizens they want to reach does matter. Policymakers should listen to and take seriously people in the welfare sector, while those people must keep up with developments and share their knowledge of robotics more widely in order to reach decision-makers.

### Limitations and suggestions for future research

The limitations of the current study include, for example, the fact that not all respondents had direct experience in the use of robots in welfare services. Positive attitudes towards robots consistently correlate with one’s degree of experience with robotic devices [e.g., 12, 48, 49]. However, this could be difficult to ensure, and of course, even if participants had had experience in using robots, it would likely have been with only one kind of robot. So, it might be easier to envision the skills needed if they are not “attached” to one particular robot; as mentioned, the HRI is naturally dependent on the type of robot. One limitation is that the number of respondents from different fields (i.e., members of parliament, ministries, enterprises in the field of robotics, associations, research institutes, and municipalities and hospital districts) were very small, which did not allow us to compare the different groups because the results might have revealed their privacy. Thus, the respondents were divided into three groups: techno-positive, techno-neutral and techno-critical. In further studies the aim is to have more respondents in the different user groups so that it is possible to make the comparison between the respondents from different fields.

Another limitation is that the research was conducted only in one country, and there are differences among the national welfare systems that could impact the findings. It might prove fruitful to investigate a wider range of industries and include case studies from abroad in future research. For example, future studies could include a controlled experiment with a robot. This could be done by dividing the respondents once again into different groups based on their opinions towards welfare services.

One suggestion for future studies arose from the data: the difference between the female and male respondents. As our data was not evenly distributed between male and female respondents, there were no differences between the sexes. However, for future studies, it would be interesting to find out whether there would be any statistically significant differences between the sexes. On a further note, also the age group could be a one possibility on how to compare the respondents. Again, our data did not show any between the different age groups, which could indicate that there are individual differences in every age group. Further studies are needed to confirm this.

## Conclusions

Continuous changes and economic pressures all contribute to the need for welfare services to adopt developments in medical information, technologies, and relationships with other (health care) systems. The purpose of this study was to investigate how to manage change in a turbulent environment such as that in welfare services. The use of robots in welfare services is a change process that should be managed properly and be supported by change agents. The results of this study suggest that the successful implementation of the use of robots requires a comprehensive plan. Change management and good communication are very important in the process. The bigger picture of change in welfare services caused by the development of robots and technology encompasses the integration of people into new opportunities, the co-creation of a future workforce, the development of new services, and practical support for people experiencing the change. According to the results, the use of robots in welfare services includes challenges and opportunities, and these should be considered when implementing robots into workplaces. The staff should also adopt innovations in different time phases, which will impact the change process. When utilizing robots, it is important that staff have the needed skills and confidence to use these robots in their daily work.
